# Commonalities and Differences among Symbiosis Islands of Three *Mesorhizobium loti* Strains

**DOI:** 10.1264/jsme2.ME12201

**Published:** 2013-05-11

**Authors:** Hiroko Kasai-Maita, Hideki Hirakawa, Yasukazu Nakamura, Takakazu Kaneko, Kumiko Miki, Jumpei Maruya, Shin Okazaki, Satoshi Tabata, Kazuhiko Saeki, Shusei Sato

**Affiliations:** 1 Department of Plant Genome Research, Kazusa DNA Research Institute, 2–6–7 Kazusa-kamatari Kisarazu, Chiba 292–0818, Japan; 2 Department of Environmental Life Sciences, Graduate School of Life Sciences, Tohoku University, 2–1–1 Katahira, Aoba-ku, Sendai, Miyagi 980–8577, Japan; 3 The Research Organization of Information and Systems, National Institute of Genetics, 1111 Yata, Mishima, Shizuoka 411–8540, Japan; 4 Faculty of Life Sciences, Kyoto Sangyo University, Motoyama, Kamigamo, Kita-ku, Kyoto, Kyoto 603–8555, Japan; 5 Department of Biological Sciences, Faculty of Science, Nara Women’s University, Kitauoyanishimachi Nara, Nara 630–8506, Japan; 6 Department of Biological Sciences, Graduate School of Science, Osaka University, 1–1 Machikaneyama, Toyonaka 560–0043, Japan

**Keywords:** Rhizobia, symbiosis island, host specificity, nodulation

## Abstract

To shed light on the breadth of the host range of *Mesorhizobium loti* strain NZP2037, we determined the sequence of the NZP2037 symbiosis island and compared it with those of strain MAFF303099 and R7A islands. The determined 533 kb sequence of NZP2037 symbiosis island, on which 504 genes were predicted, implied its integration into a phenylalanine-tRNA gene and subsequent genome rearrangement. Comparative analysis revealed that the core regions of the three symbiosis islands consisted of 165 genes. We also identified several NZP2037-specific genes with putative functions in nodulation-related events, suggesting that these genes contribute to broaden the host range of NZP2037.

Soil bacteria, collectively known as rhizobia, can infect leguminous plants to form root nodules in which rhizobia and plants exchange nitrogen and carbon, both fixed from the atmosphere. To establish this nutritional symbiosis, the two partners require multiple authentications involving signaling molecules, such as flavonoids and lipo-chitin oligosaccharides, which are important for determining the effective combination of rhizobium-legume pairing, that is, the host specificity for rhizobia (23). Most of the rhizobial genes that participate in the symbiosis are situated in specific genomic compartments, either as symbiotic plasmids with independent replication origins (3, 4, 6) or as symbiosis islands on the chromosome (13, 14).

*Mesorhizobium loti*, formerly called *Rhizobium loti*, are a group of fast-growing rhizobia associated with the agriculturally important pasture legumes of the genus *Lotus* (12). Even at the time this new species was proposed, some strains of *M. loti* were known to have broader host ranges than the type strain NZP2213. These strains can form effective nodules on some *Lotus* species, such as *Lotus pedunculatus*, and on non-*Lotus* plants on which NZP2213 cannot (11). Among the broader host-range strains, *M. loti* NZP2037, originally isolated from a nodule of *Lotus divaricatus*, is the best characterized. It is known to form effective nodules not only on *L. divaricatus* but also on *L. pedunculatus*, *L. leucocephala* and some species of *Carmichaelia*, *Ornithopus*, *Clianthus*, and *Vigna* (1, 21, 24). Nevertheless, the genomic basis of the broad host range of NZP2037 is still unclear. Comparison of the symbiosis island sequences of MAFF303099 (611 kb) (13) and R7A (502 kb) (26) revealed that the two strains share highly conserved collinear DNA regions of nearly 248 kb, with multiple deletions and insertions. The collinear region contained all the genes likely to be involved in Nod factor synthesis, nitrogen fixation, and island transfer. One marked difference is that the MAFF303099 island possesses a Type III secretion system (T3SS) whereas the R7A island possesses a Type IV secretion system (T4SS). Since the symbiotic compartments have significant roles in symbiosis, analysis of such compartments of NZP2037 should shed light on the basis of the broad host range. Here we determined the sequence of the NZP2037 symbiosis island and compared it with those of the symbiosis islands of MAFF303099 and R7A.

To determine the complete sequence of the symbiosis island of the NZP2037 genome, we used the conventional Sanger method with BAC clone basis. According to the strategy described in supplemental experimental procedures, we obtained a 694 kb continuous sequence covered by 28 BAC clones. Overall, 657 potential protein coding genes were assigned to the obtained NZP2037 genome sequence based on the method described in supplemental experimental procedures. A significant level of synteny to the MAFF303099 and R7A islands was observed in a 6 kb to 534 kb region of the obtained NZP2037 genome sequence (Fig. 1). The average GC content of this syntenic region is 59.5%. The region is interrupted by two non-syntenic regions, from 172 kb to 284 kb and from 317 kb to 389 kb, on which insertion sequences (IS) or insertion sequence fragments (ISfr) accumulate (Fig. S1), as is the case in the MAFF303099 and R7A islands. Next to this syntenic region, there is a genome region (534 kb to 654 kb) without synteny against the MAFF303099 genome, which is followed by a region (654 kb to 694 kb) syntenic to outside of the symbiosis island (5,852 kb to 5,889 kb) of the MAFF303099 genome (Fig. S1). The average GC contents in both of these two regions were 63.4%.

It is believed that the symbiosis island was inserted into a phe-tRNA gene in the genome with a 17-bp duplication of the 3′ terminal portion of the phe-tRNA gene in both the MAFF303099 and R7A genomes (25, 26). On the obtained NZP2037 sequence, the sequence identical to the 17-bp duplication in the MAFF303099 and R7A islands was found at one end of the sequence at the coordinates 6,074–6,090, but no trace of a complete phe-tRNA gene was detected in the other end region. To identify the other end of the symbiosis island, a BAC clone (NZB01N19) containing a putative orthologue of mll6433, which is located adjacent to the phe-tRNA gene in the MAFF303099 genome, was sequenced. As a result, the phe-tRNA gene with an adjacent 5 kb lower GC content region (22,217 to 26,810) was identified, which is followed by a high GC content region (1 to 22,216) with synteny against 1,982 kb to 1,961 kb of the MAFF303099 genome (Fig. S2). Based on the syntenic relationships and GC contents, it can be postulated that the symbiosis island of the NZP2037 genome was split into two: the 528 kb region of the obtained continuous sequence (a putative symbiosis island A) and the 5 kb region of NZB01N19 (a putative symbiosis island B in a genome region separated from symbiosis island A), by a large-scale genome rearrangement. Similar genome rearrangement was observed in two strains, USDA110 and USDA6, of *B. japonicum* (14, 15). Therefore, we could suppose there is a hot spot of rearrangement in the end regions of the symbiosis island, and/or that the other type of rearrangement in the symbiosis island would cause a functional defect in the symbiosis process.

In the assigned symbiosis island of NZP2037, 504 genes were identified, fewer than those in the MAFF303099 island (583 genes) and more than those in the R7A island (414 genes). Clusters of Orthologous Groups (COGs) (27) assignments for the predicted genes on the NZP2037 symbiosis island were carried out according to the strategy described in supplemental experimental procedures, and were compared to the COG classification of genes on the symbiosis islands of R7A and MAFF303099 (Table S1). Comparative analysis of the predicted gene products in the symbiosis islands of three *M. loti* strains (Supplemental experimental procedures) revealed that 165 genes were conserved in all three strains (Fig. S3). These conserved genes, including those required for Nod factor synthesis and nitrogen fixation, could be considered core regions of the symbiosis islands of *M. loti* strains. Thirty-five genes, including genes related to T4SS, were conserved only between NZP2037 and R7A, which is comparable to the number of genes conserved between R7A and MAFF303099 (33 genes) and between NZP2037 and MAFF303099 (25 genes). Total numbers of the unique genes were 279 for NZP2037, 181 for R7A, and 360 for MAFF303099. Many of these genes were clustered together in the evolutionarily flexible region of each island (Figs. 1 and S1).

As mentioned above, genes related to nodulation and nitrogen fixation were substantially conserved (Table S2). It is known that NodD protein regulates the transcription of *nod* (plus *noe* and *nol*) genes involved in the biosynthesis, modification, and transport of the Nod factor by binding to the upstream sequence, named *nod* box (16, 22). There are two copies of *nodD* genes in the NZP2037 island, as is the case in both MAFF303099 and R7A islands. Sixteen *nod* boxes were identified in the NZP2037 island (Table S3). In addition to the *nod* genes conserved in MAFF303099 and R7A islands, 6 *nod* boxes were located upstream from non-conserved genes, including *nodU* and *nodO*, as described below. Nitrogen fixation gene clusters, *nifHDKENX*, *nifB-fdxN-nifZ-fixU*, *nifSW*, *fdxB-nifQ*, and *fixABCX*, were also located in the same gene arrangement, each with a preceding sequence motif, TGT-N_10_-ACA, for transcriptional enhancement by the binding of NifA. As in the R7A and MAFF303099 islands, the NZP2037 island had two copies of *nifA* genes, *mln086* and *mln072*, in comparable locations. *fixNOQP* and *fixGHIS*, gene clusters involved in the generation of energy required for nitrogen fixation, were also conserved.

Among the genes conserved in NZP2037 and R7A but not in MAFF303099, the T4SS-related gene clusters were most conspicuous and highly syntenic (Fig. S4). The NZP2037 island possessed T4SS genes, *virD4*, *virG*, *virB1-virB11*, and *virA* (corresponding to *mln451*, *mln456*, *mln457*-*mln467*, and *mln468*, respectively). These genes exhibit about 90% amino acid identity with R7A T4SS-related genes (26). A putative *nod* box was found upstream from *virA*. Also found were two potential *vir* boxes: *vir* box 1, located in between the upstream region of *virG* and *virB1*, and *vir* box 2, upstream from *mln450-virD4*. The locations of the *cis*-elements in NZP2037 suggest that host-derived flavonoids activate NodD and subsequently activate the VirA/VirG two-component regulatory system to express T4SS machinery (17, 30) as in R7A (7, 8).

Adjacent to the *vir* genes for T4SS machinery, there were putative effector genes, some of which showed more complex relevance. One of these, *mln450*, situated between *vir* box 2 and *virD4*, encoded a protein with 89% identity to the product of *msi0061* (also called *ML063* in the database), which is a negative effector to nodulate *L. leucocephala* (7). Mln450 had a potential T4SS secretion signal sequence at the carboxyl terminus, like Msi0061 (Fig. S5) (28). Similar secretion signals were also found in the C-termini of products of two other genes, *mln452* and *mln454*. Mln454 was a 1080-residue protein with strong similarity to the C-terminal half of 700 residues of Msi0059 (also called ML061 in the database), which is also a negative effector to nodulate *L. leucocephala* (7). Notably, the region that was similar in Mln454 and Msi0059 was also similar to that in Mlr6316, located near T3SS genes in the MAFF303099 island (Fig. S6). The common possession of Mln454 homologue in NZP2037, R7A and MAFF303099, as well as a remnant of *virD* homolog in MAFF303099 might confirm the more ancestral acquisition of T4SS in the symbiosis island than T3SS, as pointed out by Sullivan *et al.* (26). Mln452, another effector candidate, is a 286-residue protein. Its 187 amino-terminal residues showed 73% identity to the T3SS effector protein (EFF46466.1) of *Xanthomonas fuscans* subsp. aurantifolii str. ICPB 10535 (19), while the remaining 99 C-terminal residues showed 73% identity to Msi0061.

To determine whether T4SS in NZP2037 is responsible for the breadth of the host range, we constructed a strain, T4KO, lacking 11 *vir* genes, *virB1-virB11* (Supplemental experimental procedures). An inoculation test with more than 10 *Lotus* species indicated that the symbiotic capacity of T4KO was essentially comparable to that of the wild-type NZP2037 in most species, including *L. pedunculatus* and *L. japonicus*, although weak alterations of nodulation capacity were observed with *L. conimbricensis* and *L. palustris* (Table S4).

These results suggested the limited contribution of T4SS to the host range among *Lotus* species and prompted us to search for other contributors in the NZP2037 island. Several nodulation-related genes were identified as candidates: *nodU* homolog (*mln006*), *nodO* homolog, and three accompanying genes (*mln028-mln031*) and *nodFEGA* (*mln054*-*mln057*), all of which accompanied the putative *nod* box sequence in each upstream region (Table S3). The former two, *nodU* and *nodO-mln029-mln030-mln031*, were found exclusively in the NZP2037 island, whereas homologs of *nodFE* were located in both the symbiosis island and the main chromosome of MAFF303099 (Fig. S7).

Since the *nodU* gene in *Rhizobium* sp. NGR234 is known to encode a 6-*O*-carbamoyl transferase to modify the side chain of the Nod factor and contributes to the nodulation of the *Leucaena* species (9, 10), the *nodU* homolog (*mln006*) under the control of *nod* box might contribute to the synthesis of an alternatively modified Nod factor. Similarly, products of *nodFEGA* are also revealed to participate in the synthesis of the Nod factor: NodF, NodE, NodG, and NodA are, respectively, an acyl carrier protein, a β-ketoacyl synthase, a 3-oxyoacyl carrier protein reductase, and an acyltransferase (5, 18). It is possible that *nodFEGA* (*mln054* to *mln057*) induced from the upstream *nod* box constitutes an enzyme system to transfer yet another acyl group for the Nod factor, whereas the two homologous *nodFE* genes without *nod* boxes in MAFF303099 might not be induced under similar conditions. Consequently, *nodU* and *nodFEGA* can broaden the host range by conferring variations on the Nod factor. It is reported that the *nodO* gene in *Rhizobium leguminosarum* biovar *viciae* encodes a Ca^2+^-binding protein secreted via a type I secretion system (T1SS) and could participate in early recognition by the legume host (2). It is also reported that, in *Rhizobium* sp. BR816, overproduction of NodO expands its host range to *Trifolium repens* (29). These results might imply that the *nodO* homolog in NZP2037 contributes to the enhancement of recognition capacity.

In this study, we determined the complete symbiosis island sequence of *M. loti* strain NZP2037 and identified the conserved regions in the three *M. loti* strains and several NZP2037-specific genes that might contribute to the breadth of the host range. The obtained sequence information is available in public DNA databases (DDBJ/Genbank/EMBL) under the following accession numbers: AP012557 for the obtained 694 kb continuous sequence including symbiosis island A, and AP012558 for NZB01N19 sequence including symbiosis island B.

## Supplementary Material



## Figures and Tables

**Fig. 1:**
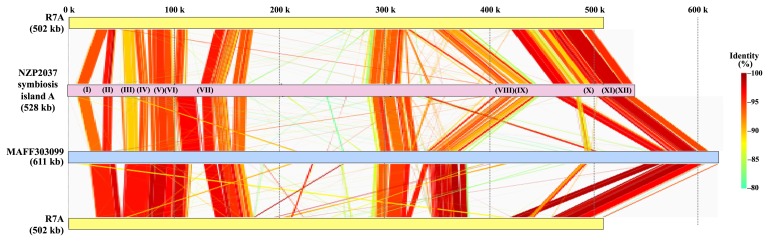
Percent identity plot of linear pairwise comparison of the symbiosis island sequences of three *M. loti* strains: R7A, NZP2037, and MAFF303099. The three *M. loti* strains were compared by GenomeMatcher programs (20). The colored horizontal bars represent the symbiosis island sequences of R7A, NZP2037 and MAFF303099. Colors indicate the percent nucleotide identity in the alignment output by BLASTN. Roman numbers on the NZP2037 bar indicate the positions of the major operons conserved in the three strains. The corresponding operons are as follows: (I): thiamine biosynthesis-related operon, (II): nodulation genes (*noeK-noeJ*), (III): biotin biosynthesis-related operon, (IV): nicotinate biosynthesis-related operon, (V): nodulation genes (*nodZ-noeL-nolK*), (VI): nitrogen fixation-related operon, (VII): nitrogen fixation-related operon, (VIII): nodulation genes (*nodS-nodA-nodC-nodI-nodJ-nolO*), (IX): nodulation genes (*nodB*, *nodD-nolL-nodD*), (X): Type IV secretion system-related operon, (XI): island transfer-related operon, (XII): nitrogen fixation-related operon
