# Supplementary material for: Commonalities and Differences among Symbiosis Islands of Three Mesorhizobium loti Strains
Source: Microbes Environ. 2013 May 11;28(2):275–8. doi: 10.1264/jsme2.ME12201 (PMC4070662; doi:10.1264/jsme2.ME12201)
Supplement: Supplementary file 1 [file 28_275_s1.pdf]

Supplementary Materials for

Commonalities and Differences among Symbiosis Islands of Three *Mesorhizobium loti* Strains

Hiroko Kasai-Maita[1, 2], Hideki Hirakawa[1], Yasukazu Nakamura[1, 3], Takakazu Kaneko[1, 4], Kumiko Miki[5], Jumpei Maruya[5,6,†], Shin Okazaki[5,‡], Satoshi Tabata[1], Kazuhiko Saeki [5], and Shusei Sato[1, 2]

Supplemental Experimental Procedures

<Sequencing of the symbiosis island of the NZP2037 genome>

BAC library construction. The genome DNA of NZP2037 was partially digested with *Mbo*I and size fractionated in the 30- to 50-kb size range by pulsed-field gel electrophoresis. The recovered DNAs were ligated with *Bam*HI digested pCC1BAC (Epicentre Bio., USA). The ligated DNAs were then used for transformation of *E. coli* EPI300 (Epicentre Bio., USA) by electroporation, and transformants were selected on LB agar plates containing 25 μg/ml chloramphenicol. A total of 3740 clones with an average insert size of 33 kb were generated and arrayed in ninety-three 384 well microtiter plates. The nucleotide sequences of both ends of the BAC clones were analyzed using a Dye-terminator Cycle Sequencing Kit and the 3730XL Sequencer (Applied Biosystems, USA).

Clone selection. Six seed clones were selected based on the end sequence information judging from the similarity to the genes located in the symbiosis island of MAFF303099, i.e. *mlr6171* (*nolO*), *mlr5907* (*nifK*), *mlr6117*, *mlr5786*, *mlr6386* (*nodM*) and *mll5867* (*nifA*). Walking clones form seed sequence were selected based on

the complete matching of the end-sequence on the seed sequence, and then confirmed by PCR using primer sets designed on the end region of the seed sequence.

DNA sequencing and data assembly. The nucleotide sequence of each BAC insert was determined according to the bridging shotgun method described previously (Sato *et al.* 1997). Briefly, the BAC DNAs were subjected to sonication followed by size-fractionation on agarose gel electrophoresis. Fractions of approximately 3.0 kb were cloned into pUC118. The plasmid DNA was amplified by TempliPhi (GE Healthcare, UK), and used as a template. Sequencing was performed using the cycle sequencing kits (Dye-terminator Cycle Sequencing kit of Applied Biosystems, USA) with DNA sequences type 3730 (Applied Biosystems, USA) according to the protocol recommended by the manufacturer. The both ends sequences, a total of which correspond to about 6 times equivalent of an insert, were assembled using Phred-Phrap programs (Phil Green, Univ. Washington, Seattle, USA). After extension of the termini of each contig by primer extension method followed by re-connection, the BAC inserts were assembled into a single contig with more than 95% coverage of either both strands or multiple reads on one strand. A lower threshold of acceptability for generation of consensus sequences was set at Phred score 20 for each base.

<Gene assignment, annotation and comparative analysis>

Gene assignment and annotation. Prediction of protein-coding regions was carried out by a combination of four prediction programs: Glimmer 3.02 (Arthur *et al.* 2007), IMC (*in silico* Molecular Cloning – In Silico Biology, Inc.), MGA (MetaGeneAnnotator) (Noguchi *et al.* 2008) and the EMBOSS getorf program (http://emboss.sourceforge.net/apps/cvs/emboss/apps/getorf.html). All of the protein-coding regions, with 120 bp or longer in length, were translated into amino acid sequences. The putative protein-encoding genes start with ATG, GTG or TTG codons.

The all predicted genes were denoted following their ordering by a serial number with the prefix ‘mln’. The putative protein-encoding genes were subjected to subsequent similarity searches against the nonredundant (nr) protein database from NCBI using the BLASTP program. Assignment of Clusters of Orthologous Groups of proteins (COGs) of predicted gene products was carried out by BLASTP analysis against the COG reference dataset (http://www.ncbi.nlm.nih.gov/COG/). A BLAST E-value of less than 10^{-4} was considered significant. After filtering, COG assignments of the putative gene products were generated according to COG identification, using the best-hit pair in the reference dataset.

Comparative analysis. Comparison of translated amino acid sequences of the assigned protein-encoding genes in three *M. loti* strains was performed by BLASTP program. The reciprocal BLAST best hit with the threshold of amino acid sequence identity ≥ 70%, the threshold of length coverage of the query sequence ≥ 80%, and a cut-off E-value ≤ 10^{-4} were considered as conserved genes.

<Interaction analysis using T4KO strain>

Deletion-insertion mutagenesis of T4SS gene cluster in *M. loti* NZP2037. An 1199-bp DNA fragment upstream from *virB1* was PCR amplified by Blend Taq DNA polymerase (Toyobo) with primers virB_KO-1, 5’-cttcaattgAAGGCATGCATCGTGAGGTAC -3’ (underlines indicate *Mfe*I and *Sph*I sites), and virB_KO-2, 5’-AGCACTCGAATTCATGCTAGCTGGGAATGACATGGATGT -3’ (*Eco*RI and *Nhe*I). The fragment was first cloned into pCR2.1-TOPO vector in reverse orientation to the *lacZα* gene to make pKMS002. The *Mfe*I-*Hin*dIII fragment was subcloned to *Eco*RI-*Hin*dIII–digested pK18mob suicide vector (Schäfer *et al.* 1994) to make plasmid pKMS005. Simultaneously, a 1772-bp DNA fragment downstream from *virB11* was

similarly amplified with primers virB_KO-3, 5'-TAGCATGAATTCGAGTGCTACGTCATCGTACCGTTCCGT-3' (*Eco*RI), and virB_KO-4, 5'- cttaagcttCGAAGCAGCGCTTAGACTTGT-3' (*Hin*dIII), and was cloned into pCR2.1-TOPO vector in reverse orientation to the *lacZα* gene to make pKMS004. pKMS004 was digested by *Eco*RI and *Hin*dIII, then inserted into *Eco*RI-*Hin*dIII-digested pKMS005 to make pKMS006. To the sole *Eco*RI site of pKMS006, a spectinomycin resistance gene (*aadA*) derived from pKST001 (Okazaki *et al*. 2010) was inserted to make T4SS knockout plasmid pKMS007. Plasmid pKSM007 was transferred into *M. loti* NZP207 by conjugation using *E. coli* S17-1. Mutants were first selected for Spc resistance and then screened for the loss of Km resistance. Mutants with the expected recombination for deletion of *virB1-virB11* were confirmed by PCR and Southern analysis. One of the confirmed mutants was named DT4SS.

Plant assays. *Lotus* plants used in this study are listed in Table 4S. Seeds were kindly provided by the Frontier Science Research Center of the University of Miyazaki, Japan; by the United States Department of Agriculture Agricultural Research Service; and by Drs. J.T. Sullivan and C.W. Ronson. For nodulation tests, seeds of *Lotus* species were scarified, surface-sterilized by immersion in concentrated sulfuric acid for 3 min, rinsed 10 times with sterile water, and germinated on 0.7% (w/v) agar plates at 24°C in the dark. After 2 to 3 days, seedlings were transferred to either agar slants made with B&D nitrogen-free medium and 0.9% agar (Broughton and Dilworth, 1971) or a plant box (CUL-JAR300; Iwaki, Tokyo, Japan) containing sterile vermiculite watered with B&D nitrogen-free medium. Inoculation of *M. loti* strains and plant cultivation were performed as described previously (Okazaki *et al*. 2010).

References for Supplemental Experimental Procedures

Arthur, L.D., A.B. Kirsten, C.P. Edwin, and L.S. Steven. 2007. Identifying bacterial genes and endosymbiont DNA with Glimmer. Bioinformatics 23: 673-679.

Broughton, W. J. and M. J. Dilworth. 1971. Control of leghemoglobin synthesis in snake beans. Biochem. J. 125:1075–1080.

Noguchi, H., T. Taniguchi, and T. Itoh. 2008. MetaGeneAnnotator: Detecting species-specific patterns of ribosomal binding site for precise gene prediction in anonymous prokaryotic and phage genomes. DNA Res. 15:387-396.

Okazaki S., S. Okabe, M. Higashi, Y. Shimoda, S. Sato, S. Tabata, M. Hashiguchi, R. Akashi, M. Göttfert, and K. Saeki K. 2010. Identification and Functional Analysis of Type III Effector Proteins in *Mesorhizobium loti*. Molecular Plant-Microbe Interactions. 23:223-234.

Sato, S., H. Kotani, Y. Nakamura T. Kaneko, E. Asamizu, M. Fukami, N. Miyajima and S. Tabata. 1997. Structural analysis *of Arabidopsis thaliana* chromosome 5. I. Sequence features of the 1.6 Mb regions covered by twenty physically assigned P1 clones. DNA Res. 4:215-230.

Schäfer, A., A. Tauch, W. Jäger, J. Kalinowski, G. Thierbach, and A. Pühler. 1994. Small mobilizable multi-purpose cloning vectors derived from the *Escherichia coli* plasmids pK18 and pK19: selection of defined deletions in the chromosome of *Corynebacterium glutamicum*. Gene 145:69–73.

Table S1. :Summary of three *M.loti* strains and these numbers of predicted genes classified into 18 Clusters of Orthologous Groups (COGs) categories

	**NZP2037**	**R7A**	**MAFF303099**
host range	broad	narrow	narrow
Isolation country	New Zealand	New Zealand	Japan
symbiosis island length [kb]	533	502	611
GC content [%]	A: 59.5, B: 57.4	59.3	59.7
Number of ORFs	504	414	583*
**Information storage and processing**	98 (19.4)	49 (11.8)	129 (22.1)
J (Translation, ribosomal structure and biogenesis)	2 (0.4)	4 (1.0)	3 (0.5)
K(Transcription)	38 (7.5)	24 (5.8)	32 (5.5)
L (DNA replication, recombination and repair)	58 (11.5)	21 (5.1)	94 (16.1)
**Cellular processes**	52 (10.3)	52 (12.6)	50 (8.6)
D (Cell division and chromosome partitioning)	0	0	0
O (Posttranslational modification, protein turnover, chaperones)	8 (1.6)	8 (1.9)	7 (1.2)
M (Cell envelope biogenesis, outer membrane)	9 (1.8)	11 (2.7)	10 (1.7)
N (Cell motility and secretion)	20 (4.0)	16 (3.9)	21 (3.6)
P (Inorganic ion transport and metabolism)	5 (1.0)	9 (2.2)	4 (0.7)
T (Signal transduction mechanisms)	10 (2.0)	8 (1.9)	8 (1.4)
**Metabolism**	159 (31.5)	129 (31.2)	187 (32.1)
C (Energy production and conversion)	29 (5.8)	25 (6.0)	29 (5.0)
G (Carbohydrate transport and metabolism)	13 (2.6)	16 (3.9)	20 (3.4)
E (Amino acid transport and metabolism)	53 (10.5)	46 (11.1)	59 (10.1)
F (Nucleotide transport and metabolism)	2 (0.4)	2 (0.5)	5 (0.9)
H (Coenzyme metabolism)	27 (5.4)	22 (5.3)	33 (5.7)
I (Lipid metabolism)	8 (1.6)	4 (1.0)	16 (2.7)
Q (Secondary metabolites biosynthesis, transport and catabolism)	27 (5.4)	14 (3.4)	25 (4.3)
**Poorly characterized**	29 (5.8)	25 (6.0)	24 (4.1)
R (General function prediction only)	23 (4.6)	18 (4.3)	19 (3.3)
S (Function unknown)	6 (1.2)	7 (1.7)	5 (0.9)
**Total**	338 (67.1)	255 (61.6)	390 (66.9)
No hits	166 (32.9)	159 (38.4)	193 (33.1)

(　): percentage of Total hits number

*One gene was newly predicted between mlr6398 and mlr6400.
The function of this gene is predicted to be the conjugal transfer protein (TrbD).

Table S2. Conserved genes involved in *nodulation and nitrogen fixation* in all three *M.loti* strains shown by their locus-tags

Gene	NZP2037	R7A	MAFF303099
Nodulation gene			
**nodSACIJ-nolO*	**mln393-mln394-mln395-mln396-mln397-mln398*	*ML0135, ML0133-ML0132-ML0131-ML0130-ML0129*	*mlr6161-mlr8755-mlr6163-mlr6164-mlr6166-mlr6171*
**nodB, nodD, *nolL, nodD*	**mln403, mln412, *mln414, mln416*	*ML0126, ML0122, ML0120 , ML0119*	*mlr6175, mll6179, mlr8757, mlr6182*
**nodM*	**mln475*	*ML0038*	*mlr6386*
**noeK-noeJ*	**mln038-mln039*	*ML0396-ML0395*	*mlr5801-mlr5802*
**nodZ-noeL-nolK*	**mln078-mln079-mln080*	*ML0366-ML0365-ML0364*	*mlr5848-mlr5849-mlr8749*
Nitrogen fixation genes			
$^{\$}$*nifHDKENX*	$^{\$}$*mln124-mln125-mln126-mln127-mln128-mln129*	*ML0303-ML0302-ML0301-ML0300-ML0299-ML0298*	*mlr5905-mlr5906-mlr5907-mlr5908-mlr5909-mlr5911*
$^{\$}$*nifB-fdxN-nifZ-fixU*	$^{\$}$*mln085-mln084-mln083-mln082*	*ML0358-ML0359-ML0360-ML061*	*mll5855-msl8750-mll5854-msl5852*
$^{\$}$*fixABCX*	$^{\$}$*mln090-mln089-mln088-mln087*	*ML0353-ML0354-ML0355-ML0356*	*mll5862-mll5861-mll5860-msl5859*
$^{\$}$*nifSW*	$^{\$}$*mln092-mln091*	*ML0351-ML0352*	*mll5865-mll5864*
$^{\$}$*fdxB-nifQ*	$^{\$}$*mln096-mln097*	*ML0347-ML0346*	*mlr5869-mlr5871*
nifA	*mln086, mln072*	*ML0357,ML0372*	*mll5857, mll5837*
fixNOQP	*mln497-mln498-mln499-mln500*	*ML0017-ML0016-ML0015-ML0014*	*mlr6411-mlr6412-msr6413-mlr6414*
fixGHIS	*mln501-mln502-mln503-mln504*	*ML0013-ML0012-ML0011-ML0010*	*mlr6415-mlr6416-mlr6417-msr6418*

* Indicates the presence of a putative nod-box as shown in Table S3.

$^{\$}$ Indicates the presence of a putative NifA-binding site

Table S3. Predicted nod boxes identified in NZP2037 symbiosis island

start	end	direction	Sequence	mismatch	Gene (position)
7651	7699	+	TATCCACCCCATGaATGCACTCAATCCAAACAATCAAaTTTACCgATCC	3	*nodU* (7811-9481)
30068	30115	+	CATCCATTTCGTAaATGTTT-CTATCGAAcaAATCGATTTccCCAgTtg	9	*nodO* (30175-31134)
39564	39612	+	TATCCATGCCATGGATGCATTCCATCCAAACAAcCAAgTTTACggATCT	4	*noeK-noeJ* (39902-42750)
53640	53687	+	TATCCATGGTATGGATGCGC-TCATCGAAACAAaCtATTTgAtCAAcCT	6	*nodFEGA* (55287-58909)
54807	54855	+	TATCCACAGCGTGGATGCGTCTTATCGAAACAAatAATTTTAtCAATCT	3	*nodFEGA* (55287-58909)
58028	58075	+	TATtCATAGCGTGGATGCAT-TCATCGAAACAAaCGATTTTgCCAATtC	5	between *nodG* and *nodA*
81755	81803	+	TATCCATAGCGTGGATGCTCGCGATCTAAACAATCAATTTTACCAATCC	0	*nodZ-noeL-nolK* (82387-85660)
324216	324263	+	TATCCATAGCGTGGATGCTC-CgATCTAAACAATCAATTTTgCCAAaCg	5	*mln311*; outer membrane protease (324354-325322)
342316	342366	+	TATCCATAGCGTGGATGCTCC-GATCTAAACAATCAATTTTgCCAATCC	2	*mln326*; putative outer membrane protease (342451-343416)
343919	343966	+	TATCCATAGCGTGGATGCTCC-GATCTAAACAATCAATTTTgCCAAaCg	4	*mln327*; putative outer membrane protease (344057-345025)
410973	411021	+	TgcttggGGCGTGGATGGCTGTCATCCAAACAATCAATTTTgCCAATCC	7	*nodS* (411332-411940)
412585	412633	+	CATCCACAGCGTGGATGCTCGCGATCTAAACAATCAATTTTACCAATCC	0	*nodACIJ-nolO* (412729-418803)
423334	423382	+	TATCCgCAAgGTGGATGCTTGTCATCGAAACAATCGATTTTgCCgATtg	6	*nodB* (423810-424469)
431174	431222	+	TATCCATAGCGTGaATGTTCGCGATCTAAACAATCGATTTTACCAAaCT	2	*nolL* (431288-433015)
497522	497570	-	CATCCATTGTtCGGATGCGTGCGATCCAAACAATCAATTTgttCAATCa	5	*virA*(494170-496728)
503603	503651	+	CATCCATGGCGTGGATGGTGGCTATCCcAACAATCAATTTTACCgAaCT	3	*nodM* (503748-505571)
Consensus in NGR234			YATCCAYNNYRYRGATGNNNNYNATCNAAACAATCRATTTTACCAATCY		

The nod box consensus sequence in NGR234 shown in van Rhijn and Vanderleyden (1995); R for either A or G; Y replacing U, T, or C; N is for any base.

Table S4. Symbiotic capacity of *virB1-virB11*deletion derivative T4KO with three *Lotus* species.

	Host plant	strain inoculated	Total nodules		Plant fresh weight (mg)		*n*
			average	SD	average	SD	
1	*L. arenarius*	wild-type	1.8	2.4	nd	nd	4
	PI631780	T4KO	0.3	0.5	nd	nd	4
2	*L. collinus*	wild-type	2.7	2.3	nd	nd	6
	PI464658	T4KO	2.5	2.6	nd	nd	6
3	*L. conimbricensis*	wild-type	19.8	4.7	168	50	9
	PI283616	T4KO	[a]14.6	4.9	190	62	9
4	*L. edulis*	wild-type	3.8	4.8	nd	nd	4
	PI244281	T4KO	2.0	3.1	nd	nd	4
5	*L. filicaulis*	wild-type	2.8	1.9	nd	nd	8
	B-37	T4KO	2.6	1.9	nd	nd	8
6	*L. glinoides*	wild-type	4.1	2.2	nd	nd	8
	PI246736	T4KO	4.1	2.2	nd	nd	8
7	*L. hybrid*	wild-type	1.7	1.0	nd	nd	6
	PI340798	T4KO	1.5	1.2	nd	nd	6
8	*L. japonicus*	wild-type	8.3	4.1	438	198	8
	B-129	T4KO	7.3	3.8	352	172	8
9	*L. japonicus*	wild-type	9.5	6.2	522	274	12
	MG-20	T4KO	8.2	4.0	362	153	12
10	*L. mearnsii*	wild-type	1.7	0.8	nd	nd	6
	PI226275	T4KO	2.7	1.8	nd	nd	6
11	*L. palustris*	wild-type	18.8	6.1	673	216	12
	PI284674	T4KO	[b]26.1	9.3	[b]871	348	12
12	*L. parviflorus*	wild-type	2.6	2.1	nd	nd	8
	PI283615	T4KO	2.9	2.1	nd	nd	8
13	*L. pedunculatus*	wild-type	6.8	3.5	489	183	10
	cultivar MAKU	T4KO	5.9	3.7	563	292	10
14	*L. pedunculatus*	wild-type	3.4	2.9	nd	nd	7
	PI631960	T4KO	2.0	2.9	nd	nd	7
15	*L. subbiflorus*	wild-type	4.4	2.4	nd	nd	8
	PI109314	T4KO	2.6	0.7	nd	nd	8
16	*L. uliginosus*	wild-type	3.3	2.9	nd	nd	8
	PI237188	T4KO	2.9	2.7	nd	nd	8

Nodule numbers and fresh weights were determined 6 to 7 weeks after inoculation of the bacterial strains.
[a] wild-type > T4KO (P≤0.05); [b] wild-type < T4KO (P≤0.05)
nd, not determined; fresh weight was determined only for model host species or hosts with nodulation phenotype significantly denpended on T4SS.

(a): The main portion of symbiosis island.

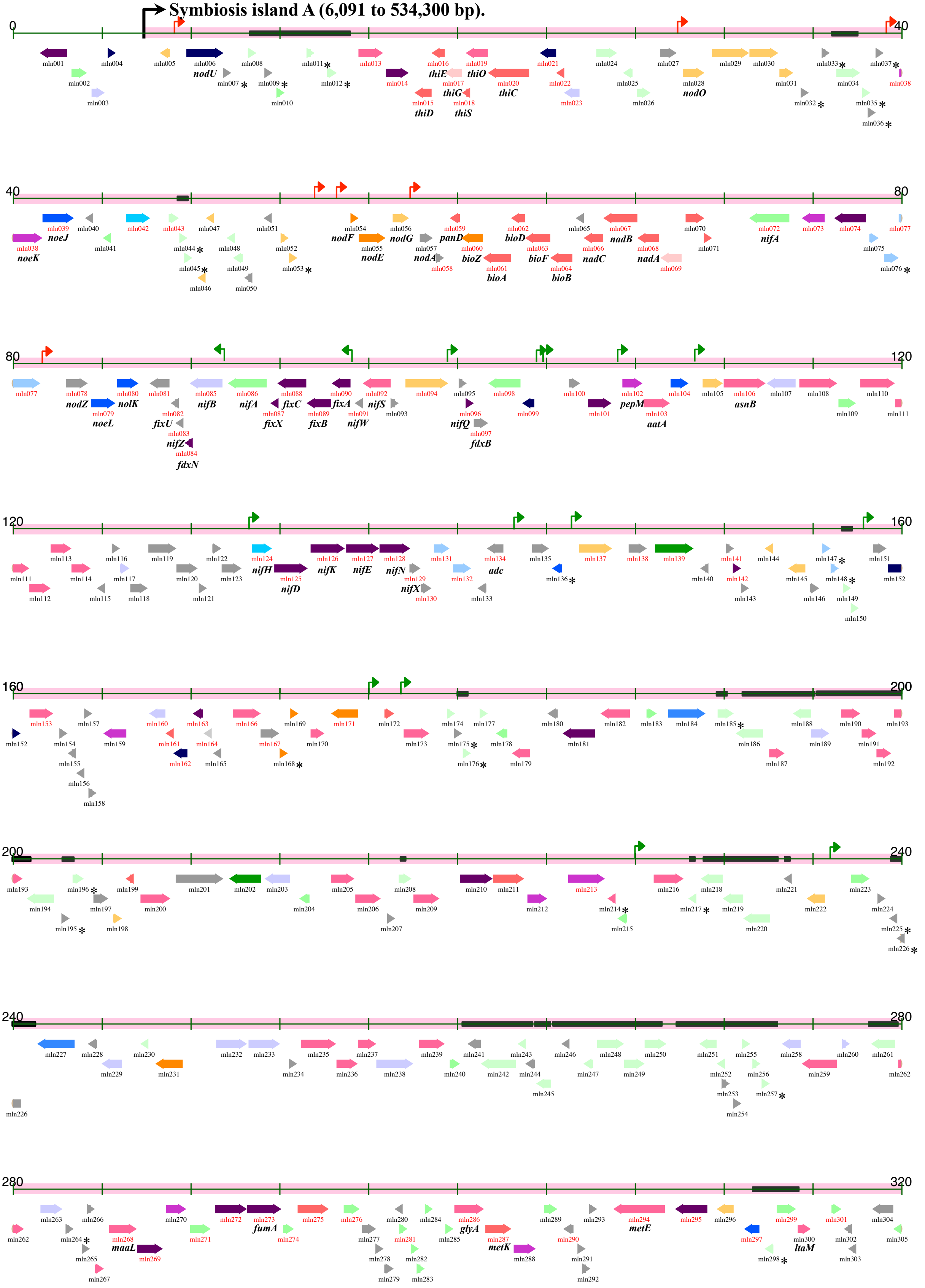

Fig. S1. (a)

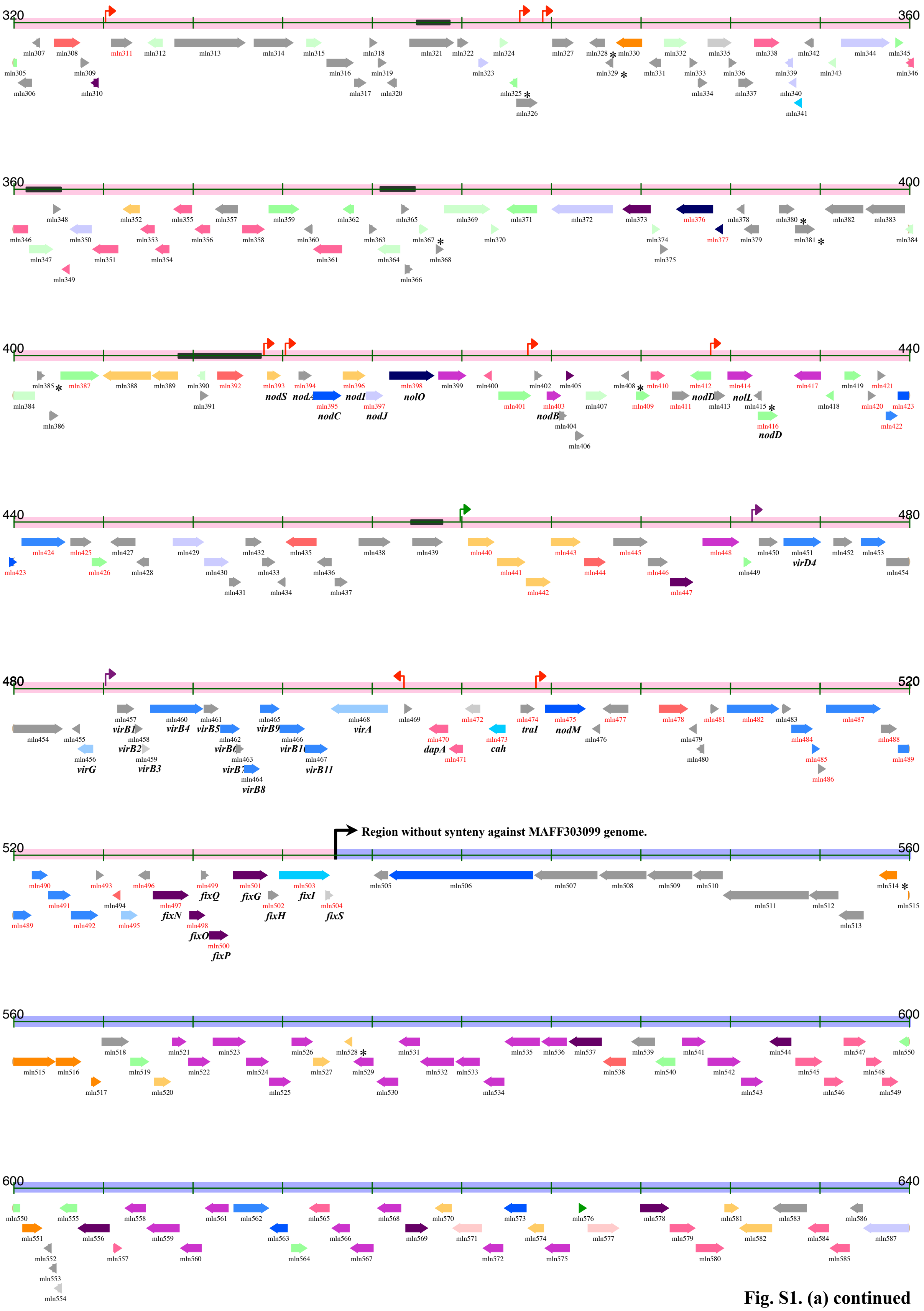

Fig. S1. (a) continued

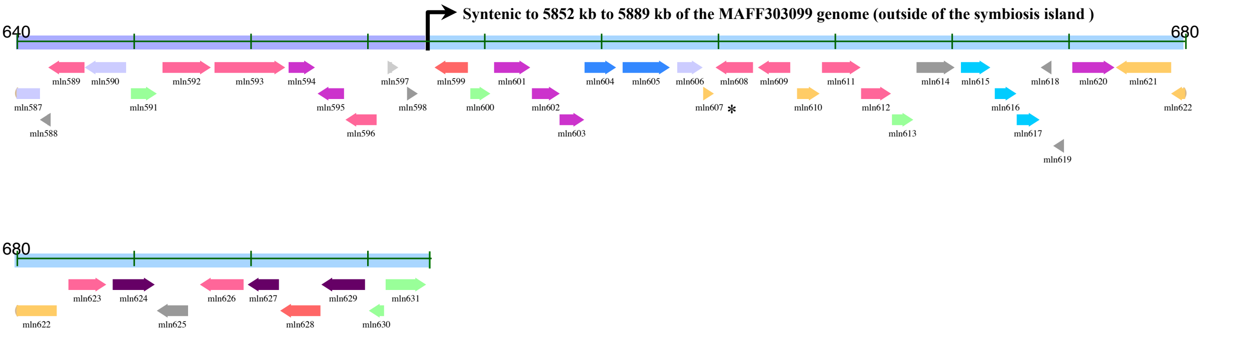

(b):The fragment of symbiosis island.

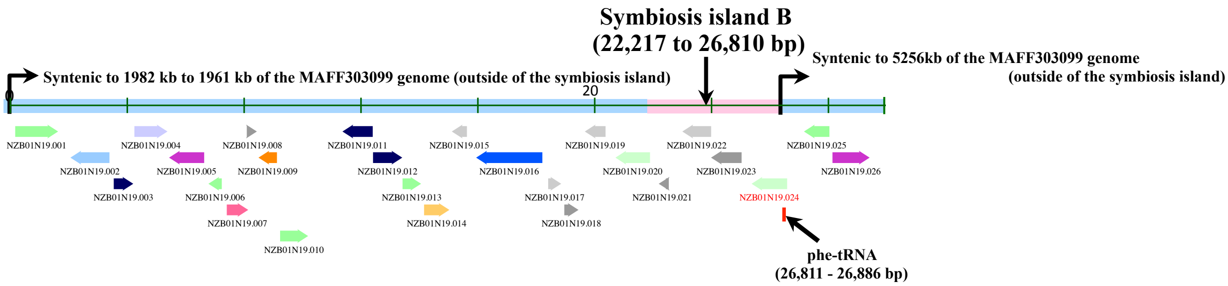

Information storage and processing

- J (Translation, ribosomal structure and biogenesis)
- K(Transcription)
- L (DNA replication, recombination and repair)

Cellular processes

- D (Cell division and chromosome partitioning): Not detected
- O (Posttranslational modification, protein turnover, chaperones)
- M (Cell envelope biogenesis, outer membrane)
- N (Cell motility and secretion)
- P (Inorganic ion transport and metabolism)
- T (Signal transduction mechanisms)

Metabolism

- C (Energy production and conversion)
- G (Carbohydrate transport and metabolism)
- E (Amino acid transport and metabolism)
- F (Nucleotide transport and metabolism)
- H (Coenzyme metabolism)
- I (Lipid metabolism)
- Q (Secondary metabolites biosynthesis, transport and catabolism)

Poorly characterized

- R (General function prediction only)
- S (Function unknown)
- No hits

Fig. S1. The gene map of the symbiosis island of NZP2037 and marginal regions. (a): Main portion of the symbiosis island (Symbiosis island A; 6,091 to 534,300 bp, indicated by the pink bar). (b): Fragment of the symbiosis island (Symbiosis island B; 22,217 to 26,810 bp, indicated by the pink bar). Green bars show the scale in 4 kb with numerals in kb. The predicted genes are indicated by boxes with arrowheads indicating the reading direction. The potential genes whose functions could be evaluated by similarity searches were classified into COG categories and are represented by the different color codes shown at the bottom of the figure. Names of the genes conserved in the symbiosis islands of three *M. loti* strains are indicated in red. Asterisk represents a putative truncated gene. Black bars represent putative insertion sequences (IS) or insertion sequence fragments (ISfr). Red arrows indicate positions of putative nod boxes (detailed information on nod boxes is shown in supplementary Table S3). Violet and green arrows indicate the positions of putative vir boxes and NifA-binding sites, respectively.

Fig. S1. (a) & (b)

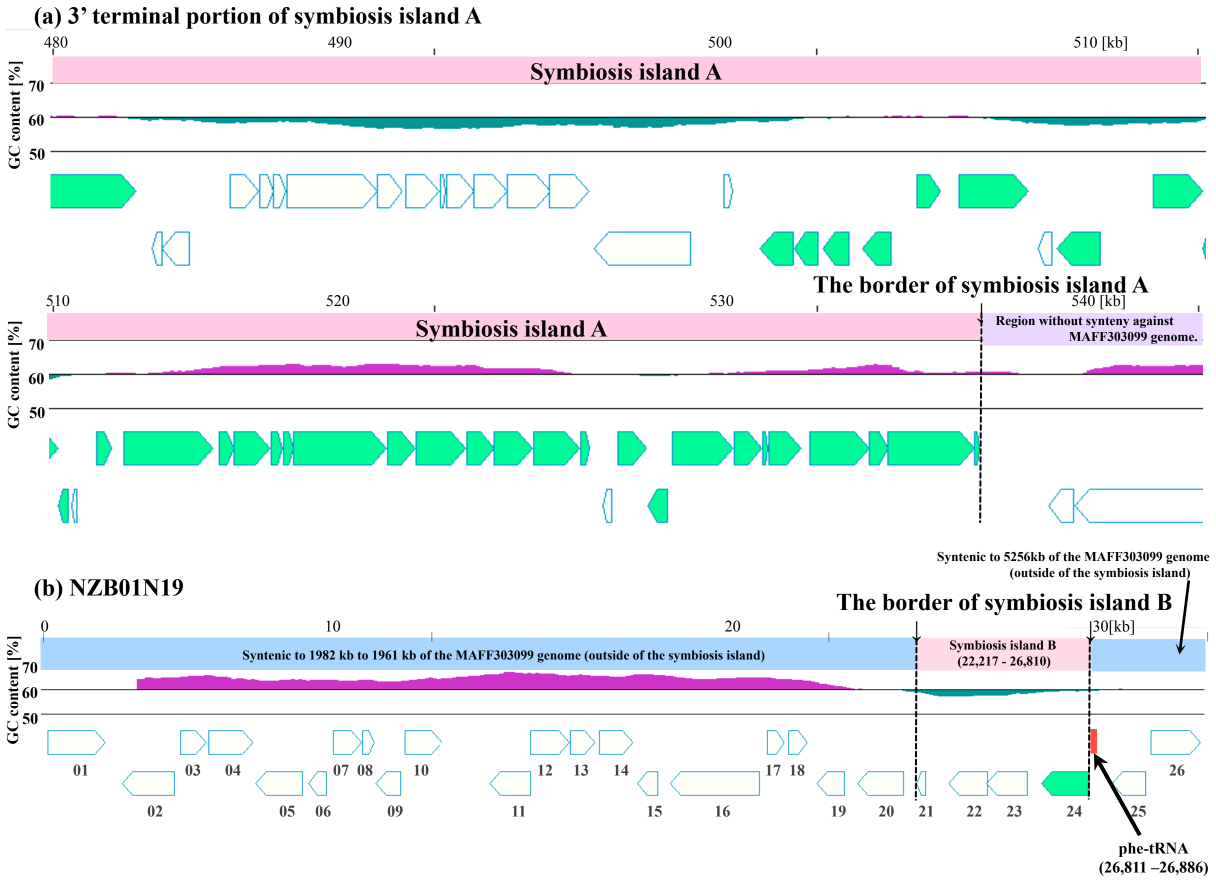

Fig. S2. The distribution of GC contents in the sequenced NZP2037 genome regions. GC contents were calculated by using a sliding window size of 5 kb with a step size of 100 bp. The GC contents of the windows are shown in magenta (≥60% GC) and green (<60% GC) bars. Predicted genes are indicated by boxes with arrowheads indicating the reading direction. Green boxes represent conserved genes in symbiosis islands of R7A and MAFF303099. (a) 3' Terminal region of symbiosis island A; the estimated border of symbiosis island A is indicated by the vertical hashed line. (b) Symbiosis island B on NZB01N19; the position of the Phe-tRNA gene is indicated by red box, and the estimated borders of symbiosis island B are indicated by the vertical hashed lines.

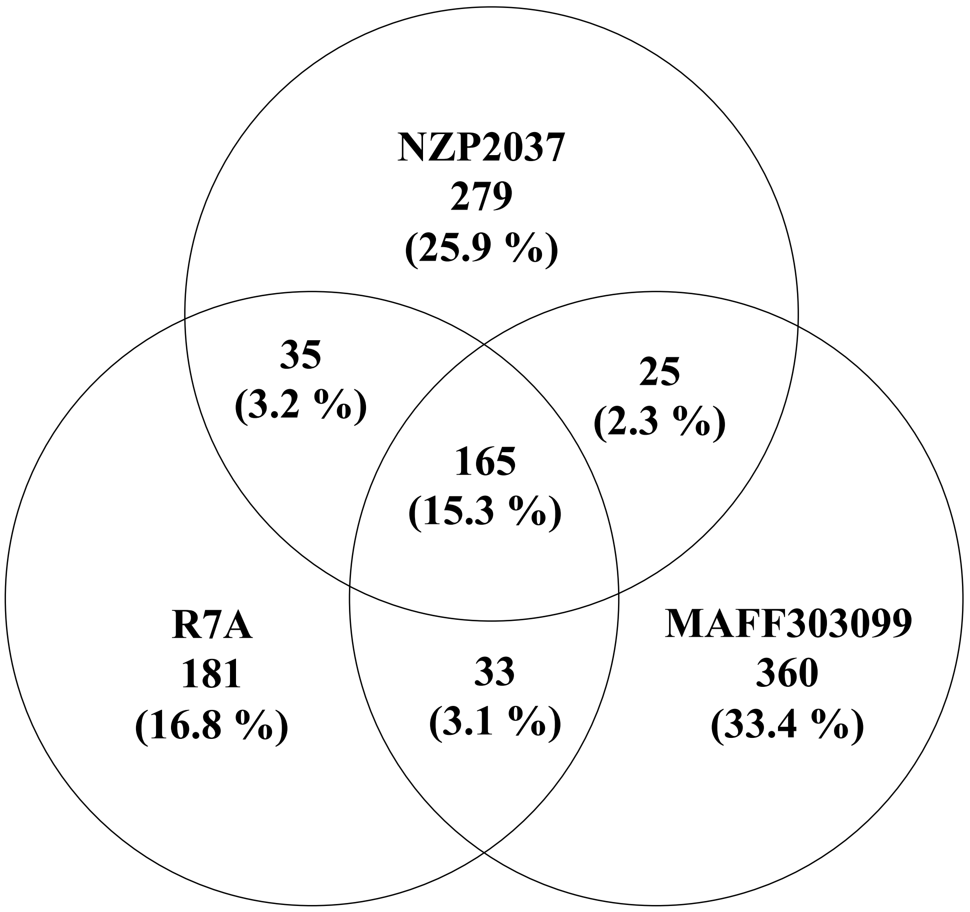

Fig. S3. Comparative orthologous gene analysis among three *M. loti* strains. The numbers of genes assigned in the symbiosis island are 504, 414, and 583 for NZP2037, R7A, and MAFF303099, respectively. The total nonredundant set of genes is 1078. The number of genes is given inside the circles representing the *M. loti* strains. The overlapping sections indicate shared numbers of genes. The proportion of the entire protein number is shown in parentheses.

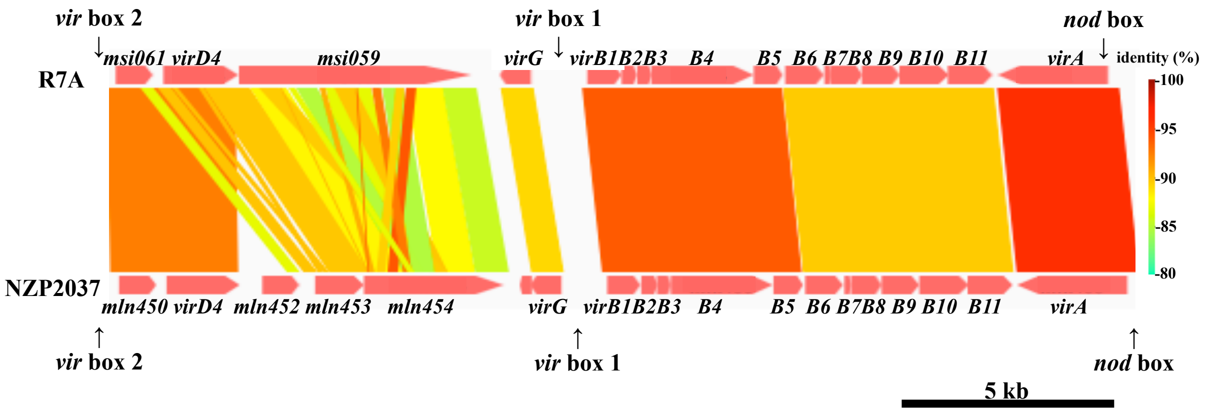

Fig. S4. Linear pairwise comparison of regions corresponding to T4SS-related genes in the islands of R7A and NZP2037. Colors indicate the percent nucleotide identity in the alignment output by BLASTN, according to the vertical scale on the right. Arrows indicate the positions of the vir box or nod box sequences in each genome.

mln450 --LEAEVRAAGAQISYEQRQRGGEIGQSLSHAYNHARE<u>REDLVAASRSRDRSGR</u>

mln452--LEAEVRAAGAQISYEQLRRGGQVGQPISHAYNHA<u>REDLMAASRSRDRSGR</u>

mln454 --KMQRTDNPQSFATGRQASEANTSSIRQDSDQT<u>RADLMASSRSRERFDAGR</u>

Fig. S5. Alignment of the C-terminal 50 amino acid sequences of three NZP2037 proteins in the T4SS region: mln450, mln452, and mln454. Arginine (R) residues in the amino acid sequences are indicated in red. Underlines indicate the consensus motif of the T4SS effector protein; in "R-X(7)-R-X-R-X-R-X-X(n)", R is R-Arginine, X is another amino acid, and the number in parentheses is the number of repetitions (30).

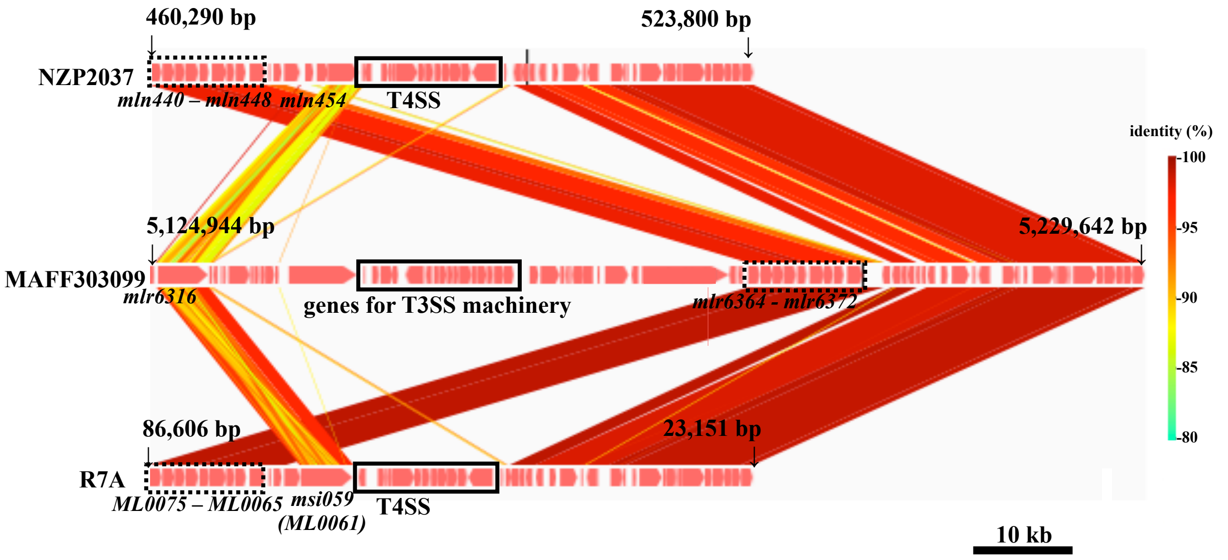

Fig. S6. Linear pairwise comparison of the genome regions surrounding the genes encoding conserved effector proteins, mln454, mlr6316, and msi059. Colors indicate the percent nucleotide identity in the alignment output by BLASTN, according to the vertical scale on the right. The black and dashed squares indicate the coding region of the secretion system and the conserved gene cluster including cytochrome P450-related genes, respectively.

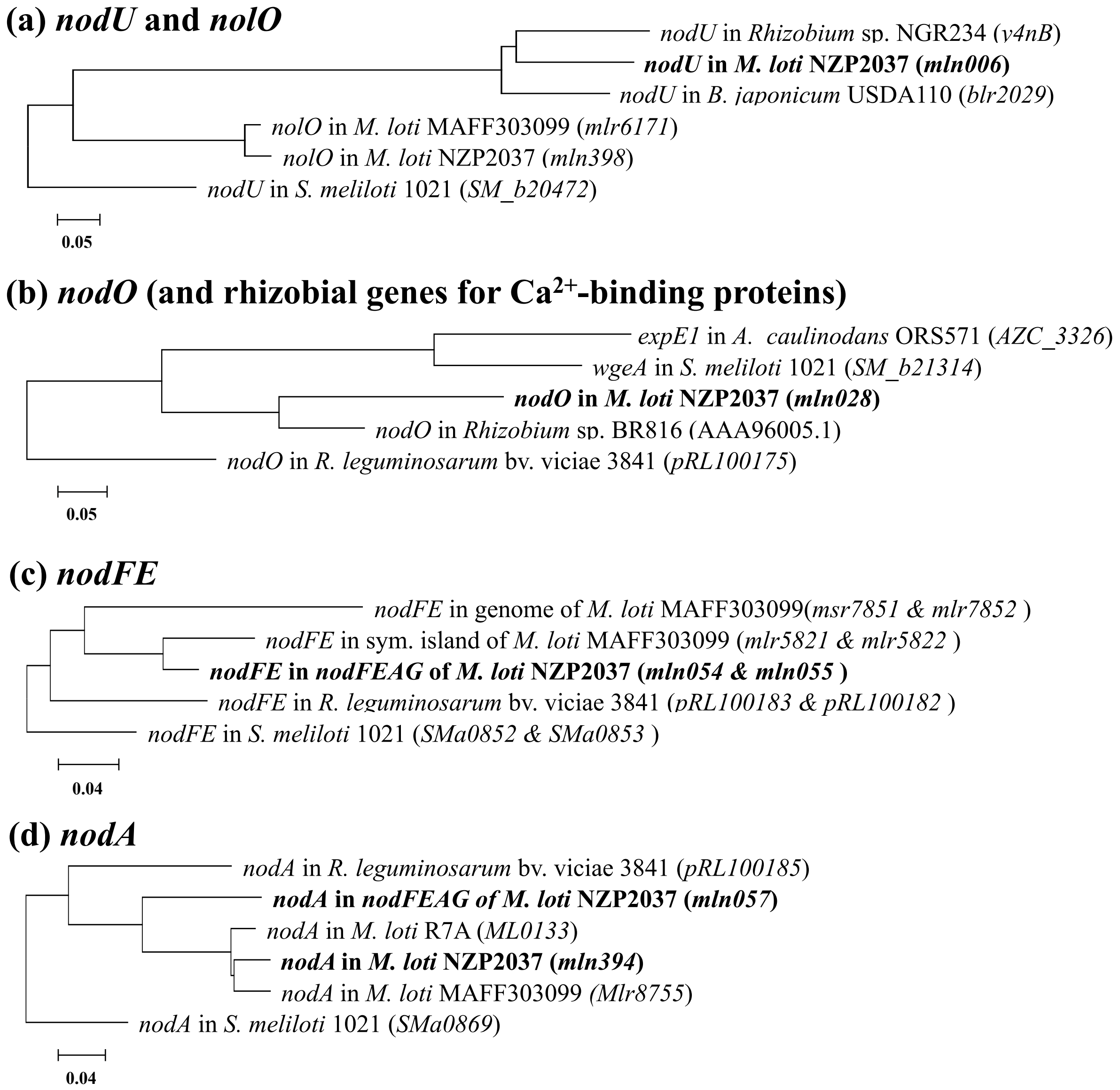

Fig. S7. Phylogenetic analysis of genes specifically detected in the NZP2037 symbiosis island and expected to contribute to the breadth of the host range. (a) *nodU* (and *nolO*), (b) *nodO* (and rhizobial genes for Ca^{2+}-binding proteins), (c) *nodFE*, and (d) *nodA*. Phylogenetic trees were constructed with MEGA ver. 5.0 by the neighbor-joining method using orthologous gene sequences of other rhizobial members (*M. loti* R7A, *M. loti* MAFF303099, *Rhizobium leguminosarum* bv. viciae 3841, *Sinorhizobium meliloti* 1021, *Rhizobium* sp. NGR234, *Bradyrhizobium japonicum* USDA110, and *Azorhizobium caulinodans* ORS571) obtained from Rhizobase (URL=http://genome.kazusa.or.jp/rhizobase/).
